# A Small Boy Makes Medical History

**DOI:** 10.3389/ti.2025.15998

**Published:** 2026-01-08

**Authors:** Reg Green

**Affiliations:** Nicholas Green Foundation, La Canada Flintridge, CA, United States

**Keywords:** bereaved families, media campaign, nicholas effect, organ donation, transplantation

Dear Editors,

In the history of medicine how many patients, thirty years after their death, have been influencing the life-and-death decisions of thousands of bereaved families around the world every year? Perhaps only one, my seven-year-old son, Nicholas, who was shot in a bungled robbery while we were on vacation in the far south of Italy in 1994 and whose organs and corneas my wife, Maggie, and I donated to seven very sick people there.

The story is so alive for millions of Italians that a new 90-min documentary called *Effetto Nicholas* on the results of that decision was recently shown by Italy’s publicly-owned RAI television channel and may soon be available in English.

Given that the shortage of donated organs is the biggest obstacle to expanding transplantation, I hope readers will find of interest some of the steps taken by a small informal group of people, ‘the Nicholas Effect’ team, unpaid and without official backing of any kind that turned a sordid crime in a remote place into a worldwide movement that pushed many millions of people toward organ donation, saved many thousands of lives and has given hope to patients everywhere.

Without them, all experience suggests the likely result would have been a spike of good resolutions to donate organs and a fairly quick return to the norm.

That this time could be different arose from our realization that although, like us, the mass of people normally give little if any thought to organ donation, the subject is potentially of interest to, literally, everyone. Anyone might need a transplant, even a world-class athlete; anyone might have to decide whether or not to donate a loved one’s organs; and anyone might be close to someone who will die without a transplant. Surprisingly then, it is in fact one of the few subjects of any kind that can command truly universal attention.

Our first conclusion from that realization was that, if presented innovatively enough, the mass media could be brought to see that their audience would find stories about transplantation riveting, not repellent. The second: the only way to reach enough people to change the donation rates significantly is through the mass media and, nowadays, their rapidly growing sibling, social media. Third, telling the story once is not enough. Like any campaign to change public opinion it has to be continuous, imaginative and adaptable.

Why is donating organs so difficult? Many reasons, but having experienced it for myself, let’s fasten on a key one. Brain death is sudden death. Families arrive at hospitals to find one of their members who was in perfect health a few hours earlier and to whom they said “Don’t be late for dinner” or “Be careful to look both ways when you cross the road” is dead or dying. Its enormity wipes out all other thoughts and even families who have been strong supporters of organ donation often falter. To this day, I remember one image that kept recurring when the doctors told us Nicholas was brain dead: never again would I hear him say “Goodnight, Daddy.”

So from the beginning we held nothing back, sharing our feelings with the world’s biggest publications and television shows -- and struck a nerve. News outlets that knew virtually nothing about organ donation -- as their questions showed -- flocked to hear more.

We also shaped the story to interest every sliver of opinion we could think of: magazines for the oldest readers (who are thrilled to know they can still do something that important when they had feared they were losing their usefulness) and the youngest (whose idealism is electrified by the thought of saving other children) women’s magazines with their huge readership, religious publications of every persuasion, sports of every kind, political groups from far left to far right and so on and on.

Reaching large audiences, we found out, is infectious. In Italy, where organ donation rates were the next to the lowest in the European Union when Nicholas was killed, they have quadrupled. Far from being horrified by the thought of diminishing the little American boy by taking away his body parts, the whole country, from Pope to peasant, thought it enhanced him. Streets, schools, parks and hiking trails have been named for him: 154 of them (!) at last count.

So by constant repetition, the hitherto scarcely believable act of removing the organs of someone already dead, putting them into people who are dying and getting several healthy people out of it became real for viewers and readers from Venezuela to Siberia. Even now, hardly a day goes by without us reminding anyone who will listen that the fate of several families could be in their hands at any time.

As the years have rolled by transplantation has shown itself to be transformative, not simply a prolongation of sickly lives: 31 years after the transplants both Nicholas’ corneal recipients are still alive, two of the organ recipients lived gratefully for 15 and 22 years before dying and the three others, though once at death’s door, are still alive. One of them has done what was once impossible, given birth to two children, the first named Nicholas -- and in a family with a history of liver disease he is fit enough to be in the Italian navy.

To urge this along I wrote two books, *The Nicholas Effect* and *The Gift that Heals*; Maggie and I were part of the team that made the Jamie Lee Curtis television movie, *Nicholas’ Gift,* that has now been seen by more than 70 million people; and we made several 12-min educational documentaries that can be downloaded free from our website^1^. Hospitals around the world have shown them to the public and for training their own medical students and nurses. Schools and colleges have included them as part of civics or biology lessons.

Saving lives is, of course, the purpose of organ donation but it has a secondary result, a demonstration that if tragedy strikes people needn't turn inward in bitterness or despair but can save on average three or four desperate strangers, the vast majority of donor families finding some solace, knowing that when the crisis of their lives hit with full force they lived up to their own highest values.

Organ donation doesn't take the pain away -- what could? - But it can give us all the strengthened hope for a better world.

## Data Availability

The original contributions presented in the study are included in the article/supplementary material, further inquiries can be directed to the corresponding author.

## Footnotes

1
http://nicholasgreen.org/

